# Techniques and short-term outcomes for total minimally invasive Ivor Lewis esophageal resection in distal esophageal and gastroesophageal junction cancers: pooled data from six European centers

**DOI:** 10.1007/s00464-016-4938-2

**Published:** 2016-04-29

**Authors:** Jennifer Straatman, Nicole van der Wielen, Grard A. P. Nieuwenhuijzen, Camiel Rosman, Josep Roig, Joris J. G. Scheepers, Miguel A. Cuesta, Misha D. P. Luyer, Mark I. van Berge Henegouwen, Frans van Workum, Suzanne S. Gisbertz, Donald L. van der Peet

**Affiliations:** 1 Department of Gastrointestinal Surgery, VU University Medical Center, Amsterdam, The Netherlands; 2 Department of Gastrointestinal Surgery, Catharina Hospital, Eindhoven, The Netherlands; 3 Department of Gastrointestinal Surgery, Canisius Wilhelmina Hospital, Nijmegen, The Netherlands; 4 Department of Gastrointestinal Surgery, Hospital Universitari Dr. Josep Trueta, Girona, Spain; 5 Department of Gastrointestinal Surgery, Reinier de Graaf Hospital, Delft, The Netherlands; 6 Department of Gastrointestinal Surgery, Academic Medical Center, Amsterdam, The Netherlands; 7 Department of Surgery, VU University Medical Center, De Boelelaan 1117, 1081 HV Amsterdam, The Netherlands

**Keywords:** Esophageal cancer, Esophagectomy, Minimally invasive, Ivor Lewis, Intrathoracic anastomosis

## Abstract

**Introduction:**

Esophagectomy for cancer can be performed in a two-stage procedure with an intrathoracic anastomosis: the Ivor Lewis esophagectomy. A growing incidence of distal and gastroesophageal junction adenocarcinomas and increasing use of minimally invasive techniques have prompted interest in this procedure. The aim of this study was to assess short-term results of minimally invasive Ivor Lewis esophagectomy (MIE-IL).

**Methods:**

A retrospective cohort study was performed from June 2007 until September 2014, including patients that underwent MIE-IL for distal esophageal and gastroesophageal junction cancer in six different hospitals in the Netherlands and Spain. Data were collected with regard to operative techniques, pathology and postoperative complications.

**Results:**

In total, 282 patients underwent MIE-IL, of which 90.2 % received neoadjuvant therapy. Anastomotic leakage was observed in 43 patients (15.2 %), of whom 13 patients (4.6 %) had empyema, necessitating thoracotomy for decortication. With an aggressive treatment of complications, the 30-day and in-hospital mortality rate was 2.1 %. An R0-resection was obtained in 92.5 % of the patients. After neoadjuvant therapy, 20.1 % of patients had a complete response.

**Conclusions:**

Minimally invasive Ivor Lewis esophagectomy for distal esophageal and gastroesophageal junction adenocarcinomas is an upcoming approach for reducing morbidity caused by laparotomy and thoracotomy. Anastomotic leakage rate is still high possibly due to technical diversity of anastomotic techniques, and a high percentage of patients treated by neoadjuvant chemoradiotherapy. An aggressive approach to complications leads to a low mortality of 2.1 %. Further improvement and standardization in the anastomotic technique are needed in order to perform a safe intrathoracic anastomosis.

In 1946, a standardized approach to esophageal resection for carcinoma of the middle third of the esophagus was introduced by Ivor Lewis [1]. This approach involved a two-stage procedure that included a laparotomy with lymphadenectomy of the celiac trunk and formation of a gastric conduit and, 1–2 weeks later, a right thoracotomy with esophageal resection, peri-esophageal and subcarinal lymphadenectomy followed by intrathoracic anastomosis. Risk of anastomotic leakage in the thorax with its potential fatal sequelae—such as empyema—resulted in the development of the three-stage approach by McKeown, with a cervical anastomosis [2]. In case of leakage, a cervical fistula remained a manageable complication [3]. While randomized evidence is limited, comparative studies suggest that cervical anastomosis is associated with less serious complications, but with more anastomotic leakage, stenosis and recurrent laryngeal nerve injuries [4]. This cervical morbidity in combination with the increased incidence of distal esophageal and gastroesophageal junction adenocarcinomas and lower postoperative morbidity after minimally invasive esophagectomy (MIE) has induced renewed interest in the two-stage procedure with an intrathoracic anastomosis [5].

In recent years, two important developments have been introduced in esophageal surgery, i.e., the systematic use of neoadjuvant treatment (chemotherapy or chemoradiation) and the implementation of minimally invasive esophagectomy (MIE) [5, 6]. Neoadjuvant treatment significantly increases 5-year survival of patients with esophageal cancer in both squamous cell and adenocarcinomas [6]. In addition, minimally invasive esophagectomy is increasingly being implemented in order to reduce postoperative respiratory complications and enhance the quality of life by avoiding a right thoracotomy and laparotomy [7–11].

Aim of this study was to assess and describe the pooled results of six European institutions performing a total minimally invasive Ivor Lewis esophagectomy (MIE-IL) with respect to technique and the resulting short-term postoperative outcomes such as morbidity and mortality and radicality of resection.

## Materials and methods

A retrospective cohort study was performed of 282 consecutive patients, who underwent a total MIE-IL for distal esophageal cancer (*n* = 160) and gastroesophageal junction cancer (*n* = 122 patients) since the introduction of this approach in June 2007 [12]. Patients originated from Girona, Spain and five centers in the Netherlands (VU University medical center and Academic Medical Center, Amsterdam; Canisius Wilhelmina hospital in Nijmegen, Catharina hospital in Eindhoven and Reinier de Graaf hospital in Delft). Data were recorded from 2007 in one center in Girona. In two centers in the Netherlands, data were recorded from 2010 and in three other Dutch centers data were recorded from 2012.

All hospitals performed at least over 20 esophagectomies per year. Two hospitals were academic hospitals, being the VU University medical center and Academic Medical Center, both located in Amsterdam. The other participating hospitals are all teaching hospitals.

A database was constructed with preoperative, intraoperative and postoperative data of these patients. The indices used were age, gender, type and location of the tumor, preoperative assessment, administration of neoadjuvant therapy, operative technique (thoracoscopic approach in prone position or left lateral decubitus position), anastomotic technique, conversions, (y)pTNM, R0 resections, circumferential resection margins, lymph node yield, postoperative complications, duration of hospital stay, intensive care (IC) stay and 30-day and in-hospital mortality.

### Statistical analysis

Continuous data were described using mean and standard deviation or median and interquartile range as appropriate for normal and non-normal distributed data. Analysis was performed with Student’s *T* test for two samples or ANOVA with post hoc analysis for *k* samples for normal distributions, and for non-normal distributions, the Mann–Whitney *U* test was applied or the Kruskal–Wallis test for *k* samples. For dichotomous and categorical data, frequencies were displayed. Analysis was performed with Chi-square and regression techniques.

### Definitions

Anastomotic leakage was defined as a full-thickness defect involving the esophagus, anastomosis or the gastric conduit and graded according to severity, in concordance with the report on standardization of data collections for complications associated with esophagectomy [13]. A grade I anastomotic leak concerned patients with a local defect, which did not require invasive therapy. A grade II anastomotic leak concerned those patients requiring interventional, but not surgical therapy (i.e., percutaneous drainage, placement of an endoscopic stent). A grade III anastomotic leak without thoracic empyema concerns those patients requiring surgical treatment such as thoracoscopic debridement, mediastinal drainage and placement of a stent, and grade IV leakage concerns established thoracic empyema requiring thoracotomy for decortication, drainage of the leakage, reconstruction of the anastomosis or resection of the necrotic gastric tube. Pulmonary complications included pneumonia as diagnosed on chest X-ray, sputum culture or CT scan and postoperative acute respiratory distress syndrome (ARDS). Wound infections were diagnosed upon positive culture or evident purulent drainage from the surgical wounds. Cardiovascular complications included atrial fibrillation, infarction or heart failure as seen on electrocardiogram (ECG), ultrasound and in laboratory findings. All complications were additionally recorded and graded according to the Clavien–Dindo classification [14].

Patients were examined clinically daily; additional examinations were performed on indication. If indicated, examination of the anastomosis consisted of computed tomography scan with oral contrast and/or endoscopy.

Postoperative monitoring for complications was similar in all included hospitals, consisting of daily assessment of clinical parameters. Upon clinical deterioration (i.e., fever, pain, tachycardia, SIRS and ileus), additional laboratory assessment and imaging were performed, consisting of CT scan imaging with oral contrast or endoscopy. Treatment was initiated immediately upon diagnosis of complications.

### Operative technique

Minimally invasive Ivor Lewis esophagectomy starts with a laparoscopic approach, in which lymphadenectomy of lymph node stations 1–4, 7–9, 11 and in some centers 12 according to the 10th edition of the JSED classification is performed [15]. Subsequently, a gastric tube is created, and a dissection of the lower paraesophageal lymph nodes via the hiatus could be performed. After changing to prone or lateral decubitus position, thoracoscopy is performed. In prone position, double lung ventilation and a pneumothorax of 6–8 mmHg are maintained with insufflation of CO_2_ with a maximum pressure of 8 mmHg. In left lateral decubitus position, the right lung is blocked and solely the left lung is ventilated. A lymphadenectomy is performed of peri-esophageal, bronchial and subcarinal lymph nodes (lymph node stations 107–111), depending on the center or on indication stations 105, 106tbL, and 106recL and 106recR according to the 10th edition of the JSED classification [16]. The esophagus is divided proximal of the arcus of the azygos vein, and before or after extraction of the specimen through a small thoracotomy, an anastomosis between the proximal esophagus and the gastric tube is performed [17].

The anastomosis is performed with different methods in the different participating centers. Some centers perform an end-to-side anastomosis using a circular stapler of 25 or 28 mm, including the 25-mm Orvil type^®^. Others perform a side-to-side anastomosis with a linear endostapler and closure of the defect with a V-Loc^®^ suture. In many cases this is followed by an omental wrap to protect the anastomosis which could reduce the sequelae of an anastomotic leakage [18]. Differences in the use of anastomotic technique reflect local expertise and the search for optimal techniques as described elsewhere [18].

In prone position, the use of a glove adhesive to the protection ring of the wound or a single port permits continuity of insufflation during formation of the anastomosis.

## Results

From June 2007 until September 2014, 282 patients in six different hospitals underwent a total MIE-IL for esophageal cancer. Each participating center performed over 20 esophagectomies per year, with two surgeons performing the procedure in each center. Pertaining characteristics of the 282 patients and peri-operative data are presented in Tables 1 and 2. In total, 90.2 % of patients received neoadjuvant therapy, usually consisting of chemoradiotherapy according to the CROSS protocol [6].

**Table 1:** Baseline characteristics of patients that underwent minimally invasive Ivor Lewis esophagectomy

Baseline characteristics	*N* (282)	%
Gender		
Male	218	77.3
Female	64	22.7
Age (mean ± SD)	62.8 ± 8.6	
Tumor type	2	
Adenocarcinoma	229	81.2
Squamous cell carcinoma	29	10.3
Adenosquamous cell carcinoma	24	8.5
Tumor location (cm) (mean ± SD)	36.2 ± 3.7	
Neoadjuvant therapy		
None	33	11.3
Chemoradiotherapy	233	82.6
Chemotherapy	16	5.7
Radiotherapy	1	0.4

Frequencies and percentages are depicted for categorical data, and mean and standard deviations (SD) are depicted for continuous data, after checking for a normal distribution

**Table 2:** Peri-operative data for minimally invasive Ivor Lewis esophagectomy

Operative data	*N* (282)	%
Type of surgery positioning	Minimally invasive Ivor Lewis esophagectomy	
Prone position	251	89.0
Lateral decubitus	31	11.0
Peri-operative blood loss (ml)	242 ± 228	
Duration of surgery (min)	333 ± 98	
Pathology		
T0	58	24.8
T1	48	20.5
T2	31	3.2
T3	94	40.2
T4	3	1.3
N0	140	59.3
N1	48	20.3
N2	34	14.2
N3	14	5.9
Number of lymph nodes	22.9 ± 9.7	
R0 resection	185	92.5
Complete regression	30	20.1
Hospital stay [median (IQR)]*		
Overall	12 (9–24)	
Uncomplicated	10 (8–13)	
Complicated	23 (12–41)	
ICU stay [median (IQR)]*		
Overall	2 (1–5)	
Uncomplicated	2 (1–3)	
Complicated	3 (1–9)	
Complications	123	43.6
Mortality (30-days)	6	2.1

*ml* milliliters, *IQR* interquartile range, *ICU* intensive care unit

* *p* < 0.001

** Mann–Whitney *U* test *p* values <0.001 regarding hospital/IC stay in uncomplicated versus complicated cases

The majority of patients (89.0 %) were operated in prone position. An end-to-side anastomosis was performed with either a 25-mm stapler in 56.3 % or a 28-mm stapler in 13.7 % of patients. A side-to-side anastomotic technique was performed in 29.4 %. An end-to-end hand-sewn anastomosis was performed in 0.7 %.

### Complications

In this cohort, four intraoperative complications were recorded. In one patient, a lesion of the splenic artery necessitated a laparoscopic splenectomy. In another patient, part of the balloon of the selective tracheal tube migrated into the right bronchus, which had to be removed by bronchoscopy. In two patients, the operation was complicated by an aortic lesion, in one patient the stapling device perforated the aortic arch, the other occurred during esophageal dissection. In both cases, the operation was converted to a thoracotomy in prone position, and both patients had an uncomplicated postoperative recovery. Conversion to an open procedure, for oncological reasons or extensive pleural adhesions, occurred in five patients (1.8 %), no mortality was reported after conversion.

An overview of all postoperative complications is depicted in Table 3. Median intensive care stay for uncomplicated patients was 2 days (IQR 1–3 days) and 3 days (IQR 1–9 days) for patients with a complicated postoperative course (all grades of complications) (*p* = <0.001). Average hospital stay for patients with an uncomplicated postoperative course was 10 days (IQR 8–13 days) as compared to 23 days (IQR 12–41 days) for patients with a complicated postoperative course (*p* = <0.001). Isolated pulmonary complications were observed in 37 patients (13.1 %), with a median hospital stay of 14 days (IQR 9.25–17 days).

**Table 3:** Postoperative complications and frequencies

Complications description	*N* (282)	%	Hospital stay median (IQR)
Anastomotic leakage	43	15.2	
Grade I	6	2.1	25 (18–40)
Grade II	8	2.8	32 (29–92)
Grade III	16	5.7	40 (25–67)
Grade IV	13	4.6	45 (37–65)
Pulmonary complications	37	13.1	
Cardiovascular complications	12	4.3	
Wound infection	9	3.5	
Bronchoesophageal fistula	2		
Other (9)			
Paraesophageal herniation	3		
Bleeding	2		
Reoperation for suspected torsion of gastric conduit	1		
Reoperation for suspected anastomotic leakage	1		
Leakage of staple line stomach	1		
Iatrogenic lesion of spleen	1		

For the different grades of anastomotic leakage, the median (IQR) hospital stay is depicted, hospital stay increases with increasing grades of anastomotic leak, with Kruskal–Wallis one-way ANOVA for *k* samples *p* < 0.001

Anastomotic leakage was observed in 43 patients (15.2 %). Using the classification proposed by Low et al., grade I leakage was observed in 6 patients (2.1 %), grade II in 8 patients (2.8 %), grade III in 16 patients (5.7 %) and grade IV in 13 patients (4.6 %) [13]. Grades and according hospital stay are depicted in Table 3. Comparison of linear versus circular stapling techniques depicted no differences in leakage rate, being 13 versus 14.9 %, respectively (*p* = 0.710).

Two patients developed a tracheoesophageal fistula following anastomotic leakage, treated with endoscopic stents in one patient and a reoperation in order to repair the fistula in the other patient. No differences were observed in leak rates between the participating hospitals (*p* = 0.334).

The effect of was determined using binary logistic regression analysis. None of the parameters were found to be predictive for anastomotic leak as depicted in Table 4.

**Table 4:** Binary logistic regression analysis for effect of anastomotic technique, duration of surgery, neoadjuvant therapy and clinical T stage on the occurrence of anastomotic leak

Parameter	*B*	Sig.	Exp(*B*)	95 % C.I. for EXP(*B*)	
				Lower	Upper
Hospital		.216			
Clinical T stage	−.512	.350	.599	.205	1.753
Neoadjuvant treatment (yes/no)	−.199	.866	.820	.081	8.281
Duration of surgery (min)	−.004	.402	.996	.987	1.005
Anastomosis (linear/circular)	−2.482	.120	.084	.004	1.916
Constant	1.126	.591	3.083		

*p* value of overall model being *p* = 0.104

Six (2.1 %) patients died in hospital, or within 30-days postoperatively, having a Clavien–Dindo grade V complication. Four patients died after developing multiorgan failure due to sepsis following pneumonia. One patient died as a consequence of heart failure. The last patient died following hemorrhage from a tracheoesophageal fistula.

### Pathology

A microscopically radical (R0) resection was obtained in 92.5 % of patients. A complete pathologic response to neoadjuvant therapy was observed in 20.1 % of patients after chemoradiotherapy. Average lymph node yield was 22.9 (±9.7) lymph nodes.

## Discussion

This multicenter pooled cohort study describes the initial results of 282 patients with distal esophageal and gastroesophageal junction adenocarcinomas, treated with neoadjuvant therapy, usually chemoradiotherapy, followed by a total minimally invasive Ivor Lewis esophagectomy [12]. The treatment resulted in a radical resection in >90 % of patients. Although the leakage rate was relatively high, extensive treatment resulted in a low combined in-hospital and 30-day mortality rate of 2.1 %, which is in concordance with other studies [5, 19]. According to these results, MIE-IL can be considered safe.

Previous studies have favored cervical anastomosis, reporting that cervical leakage was manageable with a cervical enterocutaneous fistula, with less morbidity and mortality compared to leakage following thoracic anastomosis [20]. There is evidence that cervical anastomoses are associated with a higher anastomotic leakage rate, more stenosis and more recurrent laryngeal nerve lesions, although available randomized evidence is limited [4]. A recent study showed comparable morbidity and mortality rates following cervical or thoracic anastomosis [21]. Differences in anastomotic leak rate may be explained by a shorter gastric tube segment in MIE-IL, possibly holding for better vascularization at the site of anastomosis [22].

Although our main conclusion is that MIE-IL can be considered safe, several observations should be addressed. Overall anastomotic leakage was observed in 15.2 % of patients. However, the incidence of the higher grades was relatively low. The overall leakage rate observed in this study is in concordance with the available literature [19, 23–25]. Previous studies described an empyema rate of 5 %, an anastomotic leakage requiring surgery in 4 % of patients and a gastric tube necrosis in 2 % of patients [25].

No statistically significant differences in anastomotic leak rate were observed for the five different techniques used in this cohort, although it should be noted some techniques were not applied often, and statistical power for comparison of these techniques is low. Here, the general principles of anastomosis such as tension- and rotation-free, patency and optimal perfusion are essential. Intraoperative evaluation of the anastomosis may be performed with methylene blue or endoscopy, but evidence has not been obtained in a systematic manner. The use of omentoplasty covering the anastomosis resulted in less postoperative anastomotic leakage after esophagectomy in a Cochrane study, but the outcome after transthoracic anastomosis was just not found significant probably because of the low number of studies including this type of anastomosis [26].

Regression analysis determined that anastomotic technique, duration of surgery, neoadjuvant therapy and clinical T stage were not predictive for anastomotic leak. Further emphasizing different techniques may be considered safe, depending on local expertise.

Pulmonary complications were observed in 13.3 %, similar to the results observed in the TIME-trial, in which pulmonary complications were observed in 12 % of patients in the minimally invasive group and 34 % in patients operated in the open group [5]. Interestingly, most patients in this series were operated in prone position, whereas previous series have mainly operated in lateral decubitus position [19, 25]. In the lateral approach, using selective intubation, a lung block is applied during the whole operation, whereas in prone position lung block is not necessary at all. Thoracoscopic surgery in prone position has shown to allow for earlier mobilization and less respiratory complications [27].

No recurrent nerve lesions were observed here in the present cohort. Previous studies displayed similar results with recurrent nerve lesions in 8 % of patients with cervical anastomosis and 1 % of patients with intrathoracic anastomosis [25].

Overall hospital stay and intensive care (ICU) stay were longer following postoperative complications, with an ICU stay of a median of 2 and 3 days and a median of 10 and 23 days for uncomplicated and complicated hospital stay, respectively. These results are in concordance with the literature stating average stays of 2 days for ICU and 7 days for hospital stay [25].

Indications for MIE-IL vary. Some surgeons use this approach for treating gastroesophageal junction tumors only, whereas other surgeons claim that for treating distal esophageal tumors, a safe resection can be performed with a margin of 5 cm and adequate subcarinal and paratracheal lymphadenectomy, thereby making this approach suitable for tumors located up to 5-cm distal of the carina [19, 28, 29].

Over ninety percent of patients in this cohort received neoadjuvant therapy, usually consisting of chemoradiotherapy [6]. In previous series of MIE-IL, only 29 % of patients received neoadjuvant therapy [25]. It has been stated that chemoradiotherapy might affect anastomotic healing [30]. Two series reported anastomotic complications in 6.7 and 13 % of patients that received chemoradiotherapy followed by IL esophagectomy [30, 31]. Multivariable analysis found that the preoperative radiation dose received on the fundus of the stomach was associated with anastomotic complications. The radiation dose received by the proximal esophagus was not associated with anastomotic complications following IL [30].

In the CROSS study, no differences were found in predominantly cervical anastomotic leakage rates between patients that received neoadjuvant chemoradiotherapy versus patients that received no neoadjuvant therapy [6].

In conclusion, considering the increase in distal adenocarcinomas in the West and possible benefits on postoperative morbidity, the advantages of a two-stage procedure should be recognized: a shorter gastric tube segment accounting for better vascularization of the anastomosis, less recurrent nerve injuries and less stenosis compared to cervical anastomosis. Long-term oncological safety is to be determined in follow-up studies. Future research should address implementation problems, such as standardization of operative techniques and type of anastomosis. According to the IDEAL framework, this procedure is moving from the Development to the Exploration stage. The following stage will encompass a consensus in order to select the best (two) procedure (s) in order to perform a randomized controlled trial [32]. The primary goal of the study will be to decrease all postoperative complications, while maintaining optimal quality of surgical oncological resection.
